# Weight loss and mortality risk in patients with different adiposity at diagnosis of type 2 diabetes: a longitudinal cohort study

**DOI:** 10.1038/s41387-018-0042-0

**Published:** 2018-06-01

**Authors:** Ebenezer S. Adjah Owusu, Mayukh Samanta, Jonathan E. Shaw, Azeem Majeed, Kamlesh Khunti, Sanjoy K. Paul

**Affiliations:** 1 QIMR Berghofer Medical Research Institute, Brisbane, Australia; 2 Faculty of Medicine, The University of Queensland, Brisbane, Australia; 3 Baker IDI Heart & Diabetes Institute, Melbourne, Australia; 4 Department of Primary Care and Public Health, Imperial College London, London, UK; 5 Department of Health Sciences, University of Leicester, Leicester, UK; 6 Melbourne EpiCentre, University of Melbourne and Melbourne Health, Melbourne, Australia

## Abstract

**Background:**

Undiagnosed comorbid diseases that independently lead to weight loss before type 2 diabetes mellitus (T2DM) diagnosis could explain the observed increased mortality risk in T2DM patients with normal weight.

**Objectives:**

To evaluate the impact of weight change patterns before the diagnosis of T2DM on the association between body mass index (BMI) at diagnosis and mortality risk.

**Methods:**

This was a longitudinal cohort study using 145,058 patients from UK primary care, with newly diagnosed T2DM from January 2000. Patients aged 18–70, without established disease history at diagnosis (defined as the presence of cardiovascular diseases, cancer, and renal diseases on or before diagnosis) were followed up to 2014. Longitudinal 6-monthly measures of bodyweight three years before (used to define groups of patients who lost bodyweight or not before diagnosis) and 2 years after diagnosis were obtained. The main outcome was all-cause mortality.

**Results:**

At diagnosis, mean (SD) age was 52 (12) years, 56% were male, 52% were current or ex-smokers, mean BMI was 33 kg/m^2^, and 66% were obese. Normal weight and overweight patients experienced a small but significant reduction in body weight 6 months before diagnosis. Among all categories of obese patients, consistently increasing body weight was observed within the same time window.

Among patients who did not lose body weight pre-diagnosis (*n* = 117,469), compared with the grade 1 obese, normal weight patients had 35% (95% CI of HR: 1.17, 1.55) significantly higher adjusted mortality risk. However, among patients experiencing weight loss before diagnosis (*n* = 27,589), BMI at diagnosis was not associated with mortality risk (all *p* > 0.05).

**Conclusions:**

Weight loss before the diagnosis of T2DM was not associated with the observed increased mortality risk in normal weight patients with T2DM. This emphasises the importance of addressing risk factors post diagnosis for excess mortality in this group.

## Introduction

Recent epidemiological studies have raised the controversy of the *obesity paradox* in type 2 diabetes mellitus (T2DM). While some studies reported significantly higher mortality risk in those with normal body weight at diagnosis of T2DM, compared to those with obesity^1–8^, others could not find such evidence^9,10^. Latent diseases that independently lead to weight loss before T2DM diagnosis could explain the observed increased mortality risk in those with normal weight^11^. This is particularly important, because the undiagnosed conditions leading to weight loss may also increase the risk of developing or being diagnosed with diabetes, but may be clinically diagnosed after the diagnosis of diabetes, and falsely appear as a consequence of diabetes. In this context, evaluation of weight change before and after diagnosis of diabetes along with comorbidities is crucial. However, data on these aspects at pollution level is scarce.

Only a few epidemiological studies have evaluated body weight or BMI before and after diagnosis of diabetes^12–16^. However, these studies were limited by small sample sizes^13–15^, measurement of weight at only two-time points usually many years apart^12,16^, and they did not include evaluation of the mortality risk in association with weight change. To the best of our knowledge, none of the studies that have evaluated the obesity paradox in T2DM patients conducted a dedicated analysis of body weight changes pre- and post-diagnosis of T2DM. With a large cohort of patients with incident T2DM, the aims of this real-world primary care-based longitudinal study were to evaluate:^1^ body weight changes over 3 years pre-diagnosis of T2DM^2^, body weight changes over 24 months post diagnosis of T2DM, stratified by BMI category at time of T2DM diagnosis separately for those who have died and those who have not, and^3^ the impact of weight change pattern before diagnosis on the association of BMI at time of T2DM diagnosis with mortality risk.

## Materials and methods

### Data source

The data for this retrospective longitudinal cohort study were obtained from The Health Improvement Network (THIN) database, which is a large, anonymised longitudinal dataset derived from a network of more than 600 primary care providers across the United Kingdom (UK). The THIN database is linked to other sources of hospital and national statistics data and is demographically representative of the UK. The source population includes over 13 million patients. With comprehensive patient-level longitudinal information on demographic, anthropometric, clinical and laboratory measures, clinical diagnosis of diseases and events, and prescriptions for medications, THIN database has been extensively used for academic research^17,18^. The accuracy and completeness of this database have been previously described elsewhere^19,20^. Notably, the database has a similar distribution of major chronic diseases including diabetes, heart failure, and obesity when compared to UK national statistics^4,19^. Clinically diagnosed diseases are recorded using Read codes^21–23^, and with each diagnosis, an event date is entered. Prescriptions are recorded with both British National Formulary (BNF) codes and Anatomical Therapeutic Chemical (ATC) codes along with their prescription dates.

### Participants

**Fig. 1:**
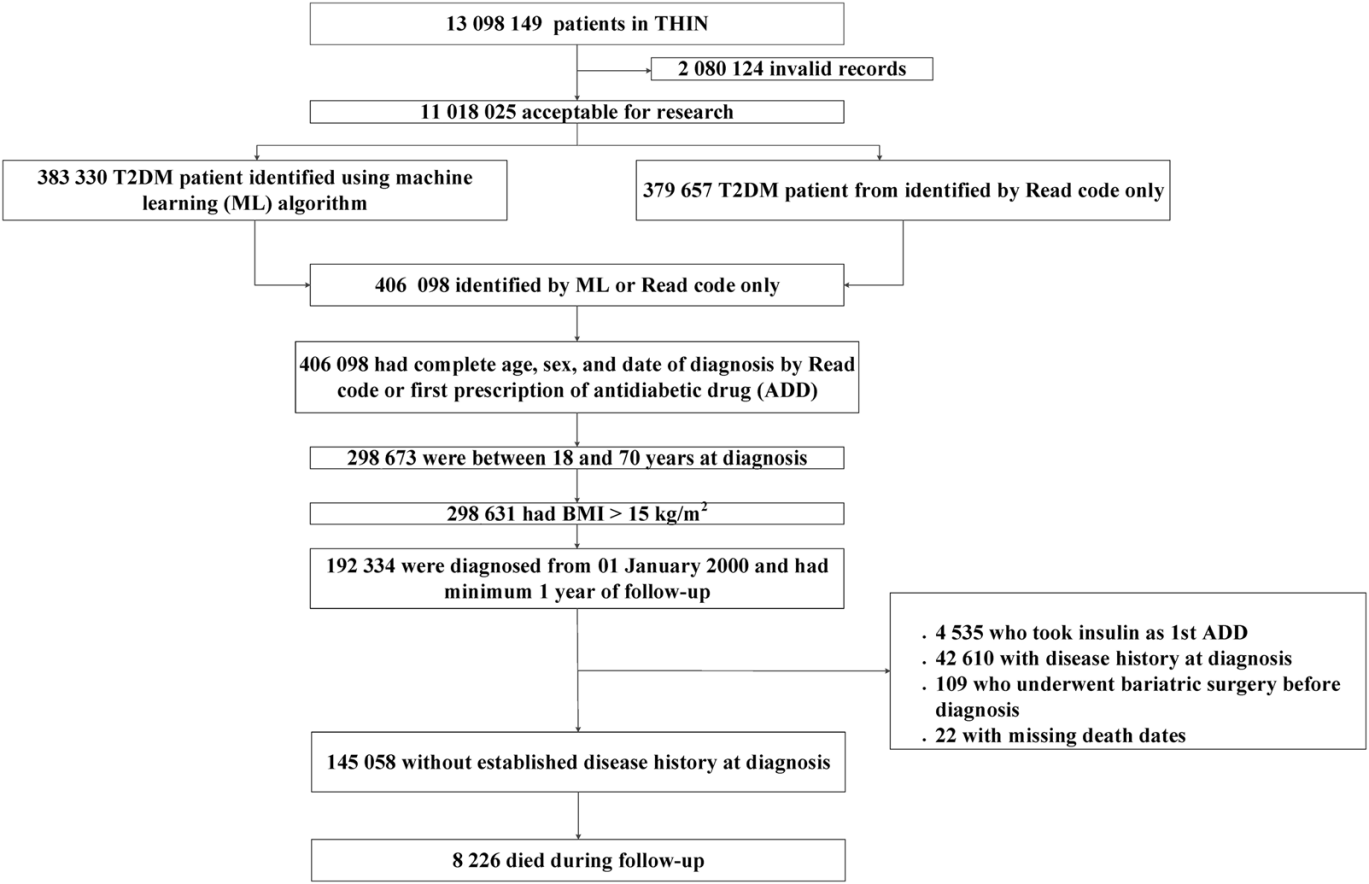
Study cohort selection flowchart

Primary care patients with T2DM were identified from Read codes or the date of the first prescription for an anti-diabetes drug (ADD), through various steps of a clinically guided iterative machine learning algorithm based on regression methodologies ^24^(See Appendix for the expanded procedure). The algorithm identified a cohort of 406,098 patients with newly diagnosed T2DM between January 1990 and September 2014. The cohort of T2DM patients for this study^1^ were newly diagnosed with T2DM from January 2000 onwards^2^, had a minimum follow-up of 1 year^3^, had complete data on age, sex, and BMI (≥15 kg/m^2^), and^4^ were without an established diagnosis of cardiovascular diseases (CVD), chronic kidney disease (CKD) or cancer at time of diagnosis of T2DM (Fig. 1). Those with Read codes for type 1 diabetes mellitus (T1DM) or gestational diabetes, those who received insulin as the first ADD, and those who had undergone bariatric surgery before or after diagnosis were excluded.

### Study variables

Patients with CVDs, CKD (any stage), and cancer with dates of diagnoses after the T2DM diagnosis date were identified using Read codes. A composite variable for CVD (any CVD) was defined as the occurrence of angina, myocardial infarction, coronary artery disease (including bypass surgery and angioplasty), heart failure, or stroke. Complete records on the prescriptions of different classes of ADDs, antihypertensive drugs, weight lowering drugs, anti-depressant drugs, and lipid-modifying drugs were extracted along with the dates of prescriptions.

Information on deaths with dates and possible cause of death were also extracted. Time to a specific disease event or death was calculated as the time from the diagnosis of T2DM to the first occurrence of the disease event or date of death respectively. Patients who were still alive at the end of the study (September 2014) or had dropped out were censored on the end date or drop out date.

Demographic, clinical, and laboratory data extracted at time of T2DM diagnosis included: smoking status, deprivation score (a socioeconomic status measure based on residential address^25^), ethnicity, body weight, BMI, glycated haemoglobin (HbA_1c_), systolic blood pressure (SBP), diastolic blood pressure (DBP), low density lipoproteins (LDL-C), high density lipoproteins (HDL-C), and triglycerides. BMI categories at diagnosis of T2DM were defined as normal weight (18.5–24.99 kg/m^2^), overweight (25–29.99 kg/m^2^), grade 1 obese (30–34.99 kg/m^2^), grade 2 obese (35–39.99 kg/m^2^), and grade 3 obese (≥40 kg/m^2^)^26^. Longitudinal measures of body weight and BMI in the 36 months before and 24 months after the T2DM diagnosis date were extracted and arranged in 6-monthly windows. All available measures on or within 3 months before the T2DM diagnosis date were considered as the baseline measures. If more than one measurement existed within this interval, the closest to the T2DM diagnosis date was taken.

Patients who lost body weight (LBW) by at least 2 kg before the diagnosis of T2DM were defined as those with weight measure in the 6 months before diabetes diagnosis was ≥2 kg less than the mean of the five possible prior measures. Those with “no weight loss” (NWL), i.e., those who remained at the same weight level or increased were also identified (see appendix for details).

The study protocol was approved by the Independent Scientific Review Committee for the THIN database (Protocol Number: 15THIN030) and the Institutional Review Board of QIMR Berghofer Medical Research Institute.

### Statistical methods

The summary statistics were presented by number (percentage), mean (SD) or median (first quartile, third quartile), and by survival status (alive or dead) where appropriate. Age-weighted rates (per 1000 person-years) for CVD, CKD, cancer, hypertension during follow-up were estimated by BMI categories and mortality status. Age-weighted mortality rates were also computed for patients under each BMI category.

Weight trajectories before and after diagnosis evaluated by fitting a generalised linear model under general estimating equations setup, with unstructured covariance. Separate analyses were conducted for each BMI category. Among patients who did not die within 2 years post diagnosis of T2DM or remained censored, the unadjusted and adjusted mean (95% confidence intervals, CI) of longitudinal 6-monthly measures of body weight before and post T2DM diagnosis were estimated respectively. Adjustment factors for post-diagnosis weight trajectory were age, sex, smoking status, the incidence of CVD, CKD or cancer, and the use of insulin, GLP-1 receptor agonists or sulfonylurea during 2 years of follow-up.

Under the hypothesis that the pattern of weight change before T2DM diagnosis could be a modifying factor on the association between BMI categories at the time of diagnosis and mortality risk, a multivariate stratified Cox regression model was fitted separately for patients under different weight loss pattern before the diagnosis of diabetes (i.e., LBW and NWL groups). Under the null hypothesis of no difference in risk patterns by BMI categories at diagnosis of T2DM, we aim to evaluate the alternative hypothesis of risk difference in patients with normal body weight compared to those with grade 1 obesity (BMI 30–34.9 kg/m^2^) at 5% level of significance. The hazard ratio (HR) for all-cause mortality was calculated for each BMI category using individuals with grade 1 obesity as the reference group. The adjustment factors were—age, sex, deprivation score, and smoking status at diagnosis; use of insulin, oral anti-diabetes drugs, and cardio-protective medications during follow-up as fixed covariates. Age groups (defined as 18–40, 41–50, 51–60, 61–70 years) at T2DM diagnosis were used as the stratification factor. Robust estimates of hazard ratios (95% CI) were obtained, and Bayesian information criteria (BIC) was used to compare the model fits. The proportional hazards assumption was assessed using scaled Schoenfeld residuals, and variables that violated the proportional hazards assumptions (incidence of cancer, any CVD or CKD during follow-up) were included in the model as time-varying covariates. All primary analyses were conducted using the imputed body weight data, with additional analyses based on complete cases for sensitivity analyses.

In sensitivity analyses for mortality, an extended model was fiited incorporating measures of HbA_1c_, SBP, LDL-C, HDL-C, and triglyceride at baseline. Other sensitivity analyses involved^1^ excluding the time-varying covariates that violated the proportionality assumption;^2^ excluding current and ex-smokers;^2^ including patients who never developed cancer^3^, possible interaction of age groups and BMI categories (stratified by weight loss patterns). Data extraction from the THIN database was conducted using SAS® 9.4 (SAS Institute), and statistical analyses were performed using STATA version 14 MP, at a 2-tailed α level of 0.05.

### Data access

Data was made are available to the corresponding author (SKP) under a licenced agreement from IMS Health UK (now IQVIA). All data access enquiries should be forwarded to Professor Sanjoy K. Paul.

### Code availability

The programming code is available from ESOA

## Results and discussion

### Cohort characteristics at diagnosis

In this cohort of 145,058 patients with incident T2DM, the mean (SD) age at diagnosis was 52^12^ years, 56% were male, 52% were current or ex-smokers, and 66% were obese. Among patients who were censored at the end of study (still alive or moved out of practice), the mean (SD) age at diagnosis was 51^12^ years with 26% aged above 60 years, 56% were men, and the proportion of patients in the normal weight, overweight, and obese categories were 7, 27, and 67% respectively. Over a median follow-up of 8 years, those who died were significantly older (mean age: 60 vs. 51 years), had a higher prevalence of current and ex-smokers (63 vs. 51%), and had a higher SBP level (mean: 144 vs. 139 mmHg) at diagnosis compared to those who were censored (Table 1). Across all BMI categories the incidence rates (per 1000 person-years) for any CVD, cancer, and CKD were significantly higher among those who died compared to those censored (Appendix Table 1, all *p* < 0.01).

**Table 1:** Baseline characteristics of patients with T2DM and without history of CVD, CKD, and cancer at the time of T2DM diagnosis, by mortality status

	Mortality status at study end date		
	Alive	Dead	All
Patients, number (%)^a^	136,832 (94)	8226 (6)	145,058 (100)
Age in years, mean (SD)^b^	51 (12)	60 (9)	52 (12)
* Age group* ^a^			
≤40 years	25,693 (19)	304 (4)	25,997 (18)
41–50 years	33,313 (24)	824 (10)	34,137 (24)
51–60 years	43,069 (32)	2420 (29)	45,489 (31)
61–70 years	34,757 (26)	4678 (57)	39,435 (27)
Male^a^	76,054 (56)	4890 (60)	80,944 (56)
Smoking status^a^			
Current smoker	28,875 (21)	2385 (29)	31,260 (22)
Ex-smoker	40,821 (30)	2805 (34)	43,626 (30)
Never smoked	66,182 (48)	2823 (34)	69,005 (48)
Townsend deprivation^a^			
Least deprived	21,542 (16)	1443 (18)	22,985 (16)
Most deprived	26,678 (20)	1400 (17)	28,078 (19)
Body weight in kg, mean (SD)^b^	93.4 (19.3)	90.4 (19.1)	93.2 (19.3)
BMI (kg/m^2^)^b^, mean (SD)	32.7 (6.3)	31.8 (6.4)	32.7 (6.3)
BMI categories^a^			
Underweight	208 (<0.1)	52 (1)	260 (<0.1)
Normal weight	9770 (7)	764 (9)	10,534 (7)
Overweight	36,404 (27)	2444 (30)	38,848 (27)
Grade 1 Obese	52,400 (39)	3159 (38)	55,559 (39)
Grade 2 Obese	22,790 (17)	1054 (13)	23,844 (16)
Grade 3 Obese	15,260 (11)	753 (9)	16,013 (11)
SBP (mm/Hg)^b^	139 (17)	144 (18)	140 (17)
SBP ≥ 140^a^	64,881 (47)	4973 (60)	69,854 (48)
HbA_1c_ (mmol/mol^b^)	69 (18.6)	68 (17.5)	69 (18.6)
HbA_1c_ ≥ 58 (mmol/mol^a^)	96,567 (70)	5956 (72)	102,523 (71)
LDL-C (mmol/L^b^)	3.26 (0.75)	3.15 (0.67)	3.23(0.75)
HDL-C (mmol/L^b^)	1.16 (0.28)	1.21 (0.31)	1.16(0.28)
Triglycerides (mmol/L^c^)	1.90 (1.50, 2.36)	1.87 (1.5, 2.29)	1.90 (1.50, 2.35)
Follow-up (years)^c^	7 (4, 11)	11 (8, 13)	8 (4, 11)

^a^*n* (%)

^b^mean (SD)

^c^median (Q1, Q3)

*BMI* Body mass index, *SPB* Systolic blood pressure, *LDL-C* Low-density lipoprotein cholesterol, *HDL-C* High-density lipoprotein cholesterol

### Weight changes before diagnosis of T2DM

**Fig. 2:**
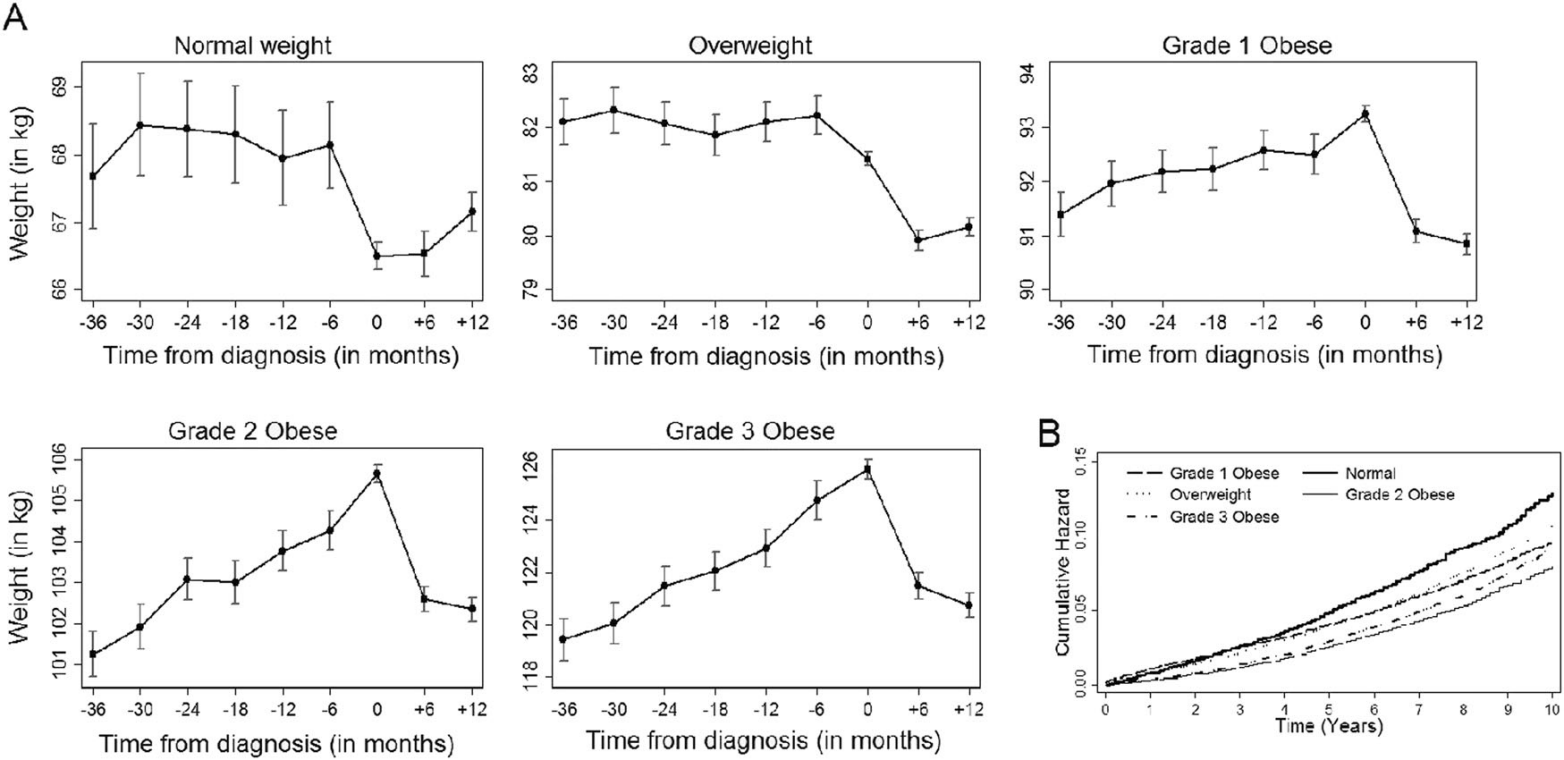
**a** Six-monthly trajectory [mean (95% CI)] of body weight (kg) over 3 years before diagnosis of T2DM, at diagnosis and 1 year post-diagnosis separately for different BMI categories at diagnosis of T2DM, for patients without history of diseases at diagnosis. **b** The cumulative hazard function for all-cause mortality in patients without disease history, by BMI categories at diagnosis

A small but significant drop in body weight during the 6 months before diagnosis of T2DM was observed in patients belonging to the normal and overweight categories at diagnosis, although a stable body weight trajectory was observed during 30 months before that time window (Fig. 2a). Among all categories of obese patients, consistently increasing body weight was observed before the diagnosis of diabetes, followed by a sharp drop in body weight during the 12 months after the diagnosis of T2DM. The proportions of patients who lost body weight in the 36 months before diagnosis date in the normal weight, overweight, grade 1 obese, grade 2 obese, and grade 3 obese categories were 28, 21, 18, 17, and 16% respectively (Table 2).

**Table 2:** Mortality risk by Body Mass Index (BMI) category at the time of diabetes diagnosis stratified by weight trajectory patterns before diagnosis

	BMI category				
	Normal weight (*N* = 10,534)	Overweight (*N* = 38,848)	Grade 1 Obese (*N* = 55,559)	Grade 2 Obese (*N* = 23,844)	Grade 3 Obese (*N* = 16,013)
Lost body weight (LBW, *n* = 27,589)					
Patients^a^	2983 (28)	8266 (21)	9721 (17)	3977 (17)	2544 (16)
Deaths^a^	205 (2)	543 (1)	520 (1)	171 (1)	120 (1)
Person-time in years^b^	1093	2957	2327	1008	667
Rate per 1000 person-years	11.0 (9.6,12.7)	11.1 (10.2,12.1)	10.6 (9.7,11.6)	8.6 (7.42, 10.1)	9.8 (8.2,11.9)
HR (95% CI)^c^	0.89 (0.65,1.22)	0.93 (0.77,1.12)	1.00 (reference)	1.01 (0.81,1.27)	1.30 (0.86,1.95)
No weight loss (NWL, *n* = 117,469)					
Patients^a^	7551 (72)	30,582 (79)	45,838 (83)	19,867 (83)	13,469 (84)
Deaths^a^	559 (5)	1901 (5)	2639 (5)	883 (4)	633 (4)
Person-time in years^b^	3069	10,981	14,443	5411	3826
Rate per 1000 person-years	12.4 (11.4, 13.5)	10.0 (9.5,10.4)	9.7 (9.3,10.1)	8.1 (7.6,8.7)	8.9 (8.2,9.7)
HR (95% CI)^c^	1.35 (1.17,1.55)	0.99 (0.91,1.07)	1.00 (reference)	0.95 (0.86,1.04)	1.06 (0.89,1.25)

^a^Number (proportion)

^b^Person-time (years) contributed by patients who died during follow-up. Follow-up period from 2000 to 2014

^c^Estimates of hazards ratios (HR) were adjusted for sex, smoking status, deprivation score, insulin, oral antidiabetic drugs, cardio-protective medicine and time varying incidence of cancer, chronic kidney disease and incidence of any cardiovascular disease using age group at baseline as stratification factor

### Weight change after diagnosis of T2DM

**Fig. 3:**
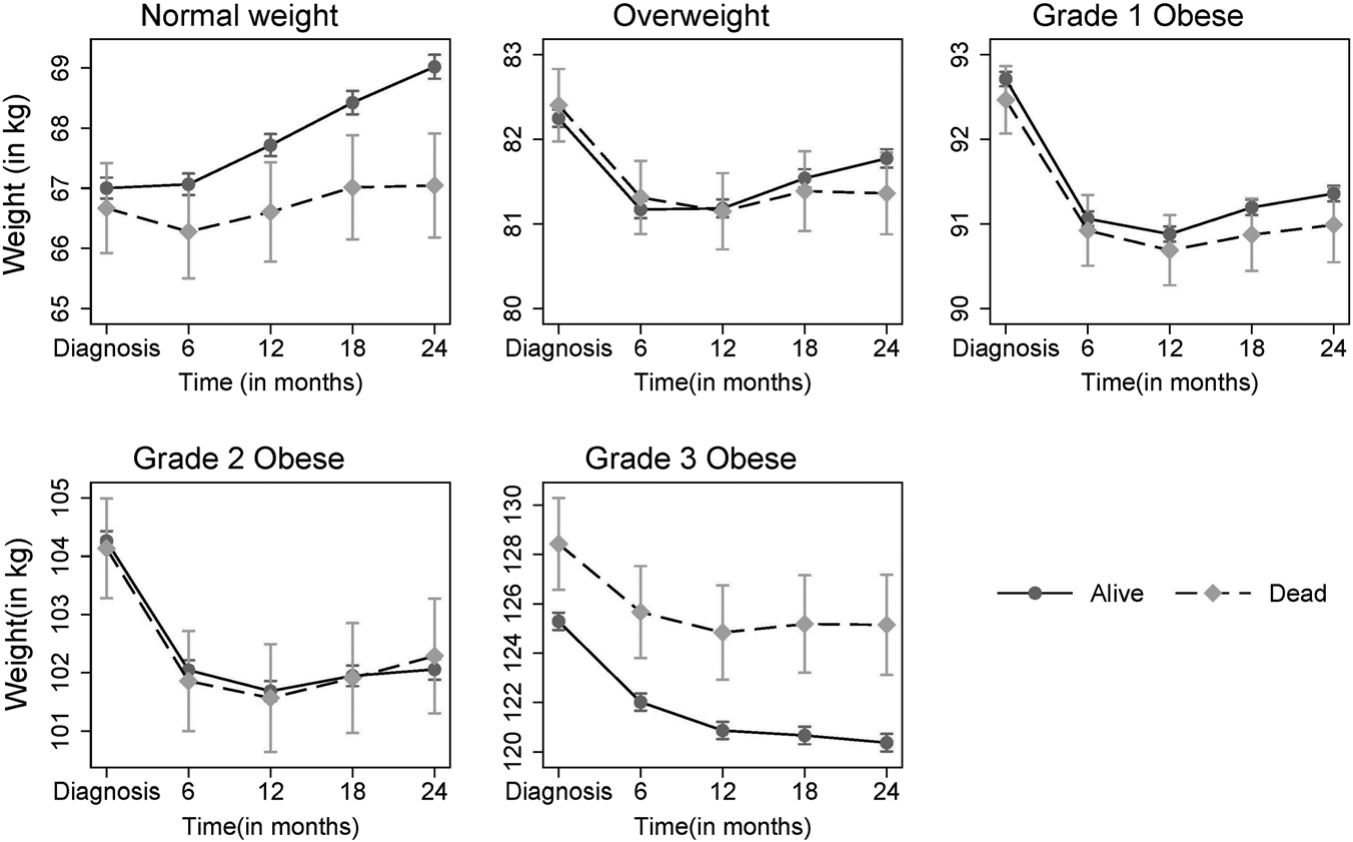
Weight (in kg) trajectory by mortality status post diagnosis of T2DM for patients without disease history. Weight trajectory estimates were adjusted for age at diagnosis, sex, smoking status, the incidence of chronic kidney disease or cancer or any CVD, and the use of insulin or sulphonylureas or GLP1RA within 2 years of diagnosis

Among normal weight patients who died, there was no indication of any weight loss during 24 months before death, while a consistently increasing body weight trajectory was observed among those who did not die (Fig. 3). With an initial significant decline in body weight within 6 months post diagnosis of diabetes, overweight, grade 1 and 2 obese patients slowly gained weight over the following 18 months, with no difference in the longitudinal patterns by mortality status. For grade 3 obesity, those who died had a higher weight throughout the post-diagnosis period than those who remained alive. The trajectories of body weight were similar for both imputed data and the complete case analyses.

### Mortality rate and risk by BMI categories

Overall, 6% of the patients died during median 8 years of follow-up (*n* = 8226, Table 1). The median follow-up time was similar among all BMI categories, and separately for patients in the NWL and LBW groups. The number, proportions, and person-time (in years) of patients who died during follow-up under different BMI categories, separately for each weight change pattern before diagnosis, are presented in Table 2. Overall, patients with normal weight had significantly increased adjusted risk of mortality compared to those with grade 1 obesity (Fig. 2b).

Among patients with NWL before diagnosis (*n* = 117,469), the age-weighted mortality rate per 1000 person-years in normal weight patients at diagnosis was significantly higher (rate = 12.4; 95% CI: 11.4, 13.5) than for those who were grade 1 obese (rate = 9.7; 95% CI: 9.3, 10.1), grade 2 obese (rate 8.1; 95% CI: 7.6, 8.7), and grade 3 obese (rate 8.9; 95% CI: 8.2, 9.7) (Table 2). With grade 1 obese patients as reference, normal weight patients in the NWL group had 35% increased risk of mortality (Adjusted HR = 1.35; 95% CI: 1.17, 1.55; *p* < 0.01).

For patients in the LBW group, mortality rate 1000 person-years in normal weight patients at diagnosis was not significantly higher (rate = 11.0; 95% CI: 9.6, 12.7) than for those who were grade 1 obese (rate = 10.6; 95% CI: 9.7, 11.6), grade 2 obese (rate = 8.6; 95% CI: 7.4, 10.1), and grade 3 obese (rate 9.8; 95% CI: 8.2, 11.9). Subsequently, there was no significant association between BMI categories and mortality risk in the LBW group (all HR *p* > 0.05) (Table 2).

### Sensitivity analyses

The mortality risk estimates were similar in subgroups of patients who did not develop cancer and those who never smoked. The extended risk analyses by incorporating HbA1c, blood pressure, and lipids at diagnosis as covariates, also revealed similar mortality risk estimates, separately for groups of patients with and without weight loss before diagnosis. Sensitivity analysis with identification of weight loss by Approach 2 (see appendix) also provided similar results. Furthermore, our estimates were not biased by inclusion of CVD, cancer, and CKD as time-varying covariates in our regression model.

Compared to grade 1 obese patients, normal weight patients in the age groups 41–50 years and 51–60 years had significantly higher mortality risk by 65% (95% CI of HR: 1.18, 2.28), 49% (95% CI of HR: 1.21, 1.83), respectively. Across all age groups, grade 2 or grade 3 obese patients did not have higher mortality risk compared to grade 1 obese patients (Appendix Table 2).

## Discussion

In this longitudinal study of a large number of incident T2DM patients from the UK, we observed:^1^ a significant drop in body weight over the 6 months before diagnosis of T2DM in normal weight and overweight patients, followed by a marginal increases in body weight post diagnosis;^2^ no significant weight change over 24 months post diagnosis among normal weight patients who died;^3^ patients with normal body weight at time of T2DM diagnosis had a significantly higher adjusted rate and risk of all-cause mortality compared to grade 1 obese patients, and this was not explained by weight loss before diagnosis^4^, a significant age and BMI interaction, with elevated mortality risk for normal weight patients aged 41–60 years; and^5^ patients with BMI ≥ 35 kg/m^2^ at diagnosis did not have significantly higher mortality risk compared to grade 1 obese patients across all age groups.

One novel aspect of this study was the evaluation of 6-monthly longitudinal changes in body weight over 24 months post diagnosis of T2DM by mortality status and BMI at diagnosis. In the normal weight category, patients had an increasing weight trajectory over 24 months irrespective of mortality status, suggesting no sudden weight loss in these patients post-diagnosis. This observation coupled with the fact that underlying comorbidities/latent diseases were not over-represented in the normal weight group contradicts the assertion of possible weight loss due to underlying diseases^11^. We observed a marginal decrease in body weight during the 6 months post diagnosis of diabetes in overweight and obese patients, followed by a plateau, similar to that observed in other studies^27,28^. In the study by Aucott and colleagues, using ~30,000 obese or overweight Scottish adults with incident diabetes, weight change was not associated with mortality risk, while 36% reduced body weight at 2 years post-diagnosis^27^. Given the adjusted trajectories of body weight by mortality status over 2 years across BMI categories in our study (Fig. 3), weight change that occurs post diagnosis rather than pre-diagnosis is likely to be associated with long-term mortality risk.

The obesity paradox in T2DM is the phenomenon whereby significantly higher mortality risk is observed among those with normal body weight at diagnosis of T2DM, compared to those with obesity. Our finding of significantly higher mortality risk in normal weight T2DM patients at the time of diagnosis corroborates other findings and contributes to the current debate on the *obesity paradox* in T2DM^2,3,5,29,30^. We report an obesity paradox regardless of disease history at diagnosis, an observation previously reported by Thomas and colleagues^5^. Some researchers have suggested that the obesity paradox could be explained by unmeasured confounders (e.g., unrecognised underlying comorbidity/latent diseases) that lead to weight loss and are therefore over-represented in the normal weight group^31–33^. By considering the weight loss pattern before the diagnosis of T2DM as a potential confounder in the relationship between adiposity status at diagnosis and mortality risk, and by undertaking separate analyses for patients with and without co-morbid disease at diagnosis, our observation is unlikely to be biased by underlying diseases. Furthermore, a detailed exploration of the patterns of weight change over 24 months post diagnosis of diabetes establishes the robustness of our finding.

Our study reveals that patients who were obese at the time of T2DM diagnosis experienced a steady rise in body weight before diagnosis, an observation which is consistent with a previous study in Pima American Indians^14^. While only two studies either statistically modelled the trajectory of body weight or evaluated one-point observed weight 10 years prior to diagnosis of T2DM, our study explored the 6-monthly trajectory of observed body weight during the 36 months prior to diagnosis, accounted for prevalence of diseases, and assessed weight change over 24 months post diagnosis of diabetes^15,34^. We also note that normal weight and overweight patients experienced significant weight loss during 6 months before diagnosis of diabetes—a rather common, yet unexplained clinical manifestation. Our study identifies patients who consistently lost body weight and patients who remained weight neutral over 3 years before clinical diagnosis of diabetes. We found that, though a significantly larger proportion of the normal weight patients lost body weight before the diagnosis of T2DM, compared to overweight, grade 1 and grade 2 obese patients, weight loss before diagnosis was not associated with increased mortality in normal weight patients.

The strengths of this longitudinal study include the large number of incident T2DM patients with 8 years of median follow-up, a nationally representative cohort, a thorough assessment of the longitudinal trajectory of body weight before and post diagnosis of T2DM, and identification of weight loss patterns and comorbid conditions before diagnosis. Clinically diagnosed T2DM patients with a diagnosis from January 2000 were selected to ensure quality of diagnosis. Age at diagnosis was also restricted to a maximum of 70 years to avoid including older patients who were already at significantly increase mortality risk. We attempted to minimise possible confounding by adjusting for several possible confounders including antidiabetic treatment, cardio-protective medications, and smoking status while evaluating the cohort with no history of major diseases at diagnosis. However, electronic health records present challenges in terms of accuracy and completeness of the required data. The limitations of this study include non-availability of complete and reliable data on ethnicity and smoking cessation during follow-up, missing body weight data during the 36 months before diagnosis of diabetes, information on diet, exercise or weight lowering medications, and the potential residual confounders as is common in observational studies. Also, there is the potential for misdiagnosis, misclassification, and miscoding of diagnostic codes in electronic medical records^35–38^. We utilised other clinical data to minimise potential misclassification of T2DM (see Appendix section). Although we excluded all T2DM patients, who received insulin as their first anti-diabetes drug from our study cohort, some patients with T1DM might still be misclassified as having T2DM.

In conclusion, weight loss before the diagnosis of T2DM occurred independently of established severe disease conditions and was not associated with the observed increased mortality risk in normal weight patients with T2DM. While the cause of this excess mortality in T2DM who were normal weight at diagnosis remains unclear, it may reflect differences in the aetiology of diabetes in normal weight people and emphasises the importance of addressing risk factors for excess mortality in this group.
